# Exploring the Effects of Robertsonian Translocation 1/29 (Rob (1;29)) on Genetic Diversity in Minor Breeds of Spanish Berrenda Cattle via Genome-Wide Analysis

**DOI:** 10.3390/ani14050793

**Published:** 2024-03-04

**Authors:** Rafael González-Cano, Ana González-Martínez, Manuel Ramón, Miriam González Serrano, Miguel Moreno Millán, Alejandro Rubio de Juan, Evangelina Rodero Serrano

**Affiliations:** 1 Ministry of Agriculture, Fisheries and Food, Paseo Infanta Isabel 1, 28014 Madrid, Spain; rgonzalez@mapa.es; 2 Regional Center of Animal Breeding and Reproduction (CERSYRA-IRIAF), Avenida del Vino 10, 13300 Ciudad Real, Spain; arjuan@jccm.es; 3 Department of Animal Production, Faculty of Veterinary Sciences, University of Cordoba, 14071 Córdoba, Spain; agmartinez@uco.es (A.G.-M.); miriam_g92@hotmail.com (M.G.S.); pa1rosee@uco.es (E.R.S.); 4 Department of Animal Breeding and Genetics, National Institute for Agricultural and Food Research and Technology (INIA-CSIC), 28040 Madrid, Spain; 5 Department of Genetic, Faculty of Veterinary Sciences, University of Cordoba, 14071 Córdoba, Spain; ge1momim@uco.es

**Keywords:** beef cattle, diversity conservation, SNP, cytogenetic analysis, Robertsonian translocation

## Abstract

**Simple Summary:**

The Spanish cattle breeds “Berrenda en Colorado” and “Berrenda en Negro” are considered endangered breeds. For years, breeders’ associations have been implementing breeding strategies in herds to address the presence of the Robertsonian translocation (rob (1;29)), which is a disease that causes a decline in reproductive performance. Single-nucleotide polymorphism (SNP) genotyping detection techniques are presented as an appropriate means to evaluate genetic diversity. However, previous research on genetic variability in both breeds has been carried out using DNA microsatellites. Therefore, we studied the genetic diversity, population structure, and potential genetic differences among individuals of both Berrenda breeds and groups based on the presence of the Robertsonian chromosomal translocation, rob (1;29). The genetic diversity in terms of expected heterozygosity was significantly lower in those subpopulations containing rob (1;29) within the breed, but in general, the four subpopulations considered showed minor genetic differences. The presence of this Robertsonian translocation did not result in sub-structuring associated with this chromosomal disorder within either of the breeds. The improvement of the reproductive performance of the Berrenda breeds requires the implementation of breeding strategies based on the contributions to the populations of the breeding stock carrying rob (1;29).

**Abstract:**

Most of the previous studies on the genetic variability in Spanish “Berrenda” breeds have been carried out using DNA microsatellites. The present work aimed to estimate the genetic diversity, population structure, and potential genetic differences among individuals of both Berrenda breeds and groups based on the presence of the Robertsonian chromosomal translocation, rob (1;29). A total of 373 samples from animals belonging to the two breeds, including 169 cases diagnosed as rob (1;29)-positive, were genotyped using an SNP50K chip. The genetic diversity at the breed level did not show significant differences, but it was significantly lower in those subpopulations containing the rob (1;29). Runs of homozygosity identified a region of homozygosity on chromosome 6, where the *KIT* (*KIT* proto-oncogene, receptor tyrosine kinase) gene, which determines the typical spotted coat pattern in both breeds, is located. The four subpopulations considered showed minor genetic differences. The regions of the genome that most determined the differences between the breeds were observed on chromosomes 4, 6, 18, and 22. The presence of this Robertsonian translocation did not result in sub-structuring within each of the breeds considered. To improve the reproductive performance of Berrenda breeds, it would be necessary to implement strategies considering the involvement of potential breeding stock carrying rob (1;29).

## 1. Introduction

**Figure 1:**
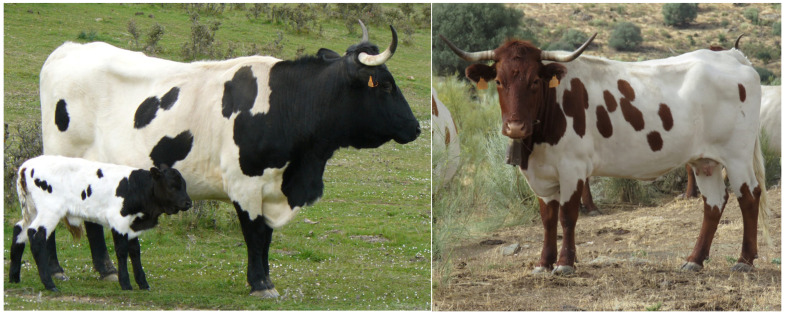
“Berrenda en Negro” (**left**) and “Berrenda en Colorado” (**right**) cattle breeds.

The Spanish Berrenda breeds, “Berrenda en Negro” (BN) and “Berrenda en Colorado” (BC) (Figure 1), are considered endangered native breeds [1] due to the decreasing number of purebred animals registered in the herd books. Regarding the number of breeding animals registered in the herd books, BN and BC have a total of 3509 (3.061 females, 448 males and 73 herds) and 6414 (5.390 females, 1.024 males and 140 herds) registrations, respectively, with most of them geographically located in the southern part of the Iberian Peninsula [2]. The effective population sizes calculated from pedigree data are 12.44 (BN) and 16.62 (BC), respectively [3].

The Berrenda breeds are valuable animal genetic resources due to their cultural, social, and environmental implications [4]. Their unique traits adapted to the Dehesa ecosystem make them the ideal choice for meat production in extensive livestock farming sites of high environmental value, such as those belonging to the Natura 2000 Network [5]. Although they are not considered efficient breeds in terms of meat production, they are resilient and able to adapt to harsh environments, which allows them to be used as maternal breeds in crossbreeding with beef cattle (Charolais and Limousin breeds). In addition, the Berrenda breeds are an important part of the Spanish cultural heritage. On one hand, they are used as draft animals, pulling carts in popular religious events. On the other hand, they are essential for the handling of fighting bulls, both in extensive farms and in bullfights, because BN and BC oxen are easily trained by farmers and also because they are larger than fighting bulls, causing them to behave as herd leaders, which facilitates the management of fighting bull herds in farms and bullrings [4].

Considering all the Spanish native breeds, the Berrenda breeds are of great interest, not only as they are considered ancestral breeds because of their phenotypic distinctiveness but also because they serve as genetic reservoirs of unique genes [4] that contribute to neutral genetic diversity [6]. The Robertsonian chromosomal translocation affecting chromosomes 1 and 29, rob (1;29), was first described in 1964 by Gustavsson [7,8]. It is so widespread that it has been identified in more than 50 cattle breeds worldwide [9,10,11]. Among Spanish cattle breeds, it has been detected in the “Retinta” breed [12] and in other threatened Andalusian breeds [13]. The consequences of using breeding stock carrying the Robertsonian translocation rob (1;29) for reproductive performance are relevant, leading to losses of fertility in both sexes [12]. On one hand, carrier females are characterized by an increase in the embryonic mortality rate, while carrier sires show a decrease in fertility [14,15].

For more than 20 years, the Berrenda en Negro and Colorado Breeders Association [16] has been developing corresponding breeding programs in both breeds, including the compilation of pedigree records and the detection of chromosomal alterations in animals that are candidates for breeding. This is aimed at improving the low reproductive performance in the two breeds [3,17], which restrains their conservation potential. Candidates for breeding stock are genetically tested to eliminate rob (1;29) carriers. Consequently, the prevalence of rob (1;29) has declined significantly in recent years. From 2009 to 2019, the prevalence of this genetic disorder decreased from 18 to 8% in BC; in the case of BN, its prevalence decreased from 47 to 11% [17].

Considering that such an intense selective strategy could negatively affect the genetic diversity accumulated in the Berrenda breeds, it is necessary to evaluate whether the implementation of selective actions against this chromosomal disorder affects both the genetic structure and the conservation potential in these two cattle populations as well as the genetic variability of the populations carrying this disorder. Significant differences in the frequency of rob (1;29) between herds have been observed in these two endangered breeds, which could be the result of reproductive isolation, uncontrolled selection, or genetic drift [13].

According to the FAO [18], the single-nucleotide polymorphism (SNP) genotyping detection techniques are an appropriate means to evaluate genetic diversity, the structure of populations, and the relationships among populations in farm animal genetic resources [19]. Compared to that of other genetic markers (such as microsatellites), SNP analysis shows lower confidence intervals, which results in an improvement in the measurement of genetic diversity, differentiation among populations, and the appropriate separation of individuals into different groups [20,21]. Moreover, SNP analysis allows more complex issues to be addressed, such as the identification of genomic regions related to economic traits, local environmental adaptations, or traces of selection and evolution [20,21,22]. The availability of new computing tools dedicated to maximizing populations’ heterozygosity using genomic data allows for the implementation of mating programs in a more effective manner than those based on pedigree records [23].

Most of the previous studies on the genetic variability in the BN and BC breeds have been carried out using DNA microsatellites [6,24,25]. In fact, genetic variability analysis in these two breeds has never been performed based on SNPs. Genetic diversity studies in cattle linked to the rob (1;29) disorder based on SNP analysis or genome-wide prediction (GWP) methods are scarce [26] and have never been performed in threatened breeds.

Therefore, the aim of this study was to analyze a large sample of BN and BC populations, differentiating between rob (1;29) carriers and animals free of this genetic disorder, by using a commercial SNP50K chip to estimate the genetic diversity and the current structure of the populations as well as the genetic relationships in each of these two Spanish native cattle breeds.

## 2. Materials and Methods

### 2.1. Sampling for the Identification of the Robertsonian Translocation Rob (1;29)

**Figure 2:**
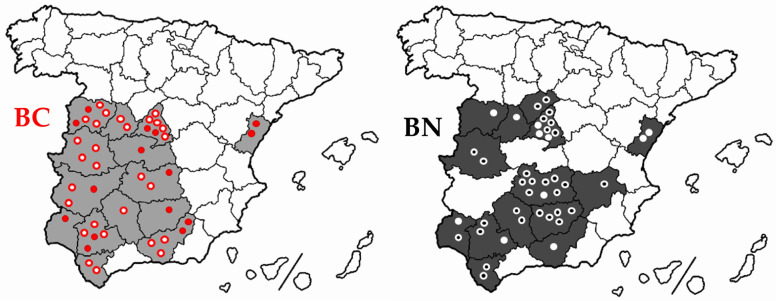
Map with the locations of the farms analyzed for the presence of rob (1;29) in the Berrenda en Colorado (*n* = 42) and Berrenda en Negro (*n* = 43) cattle breeds within the current geographical distribution areas of these breeds. Double circles identify those farms where rob (1;29) carriers were identified.

**Figure 3:**
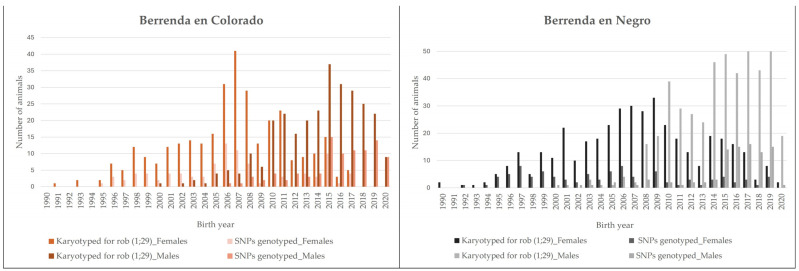
Number of animals karyotyped for rob (1;29) and SNP-genotyped animals of the breeds Berrenda en Colorado (BC) and Berrenda en Negro by birth year.

A total of 375 animals from 78 farms were sampled (Table 1 and Figure 2) from 2007 to 2022 with a birth year from 1990 to 2020 (Figure 3). All the sampled females were breeders, while calf candidates for future breeding were also included among the males. SNP genotyping was carried out on 373 samples (187 females and 186 males). Previously, all individuals had been analyzed to diagnose rob (1;29). The results showed that 204 animals were free of rob (1;29) and 169 animals were carriers of this genetic disorder (160 heterozygous and 9 homozygous carriers). All sampled animals were considered purebred as they were registered in the corresponding stud books of each of the Berrenda cattle breeds, which are both managed by the National Group of the Berrenda en Negro and the Berrenda en Colorado Breeders Association (ANABE, https://anabe.webgescan.com/, accessed on 1 March 2024). All the samples were collected in compliance with the ethical guidelines of ANABE and the University of Córdoba.

The incidence of rob (1;29) in both Berrenda breeds was different depending on the herd and independent of the geographical distribution [13]. Therefore, the sampling for this study was carried out by avoiding the possible effects of these two factors (Figure 2) and, at the same time, ensuring that both rob (1;29) carriers and non-carriers were available to be genotyped.

### 2.2. Chromosomal Analyses

Chromosomal analyses were carried out at the Laboratory of Animal Applied Cytogenetics at the University of Córdoba (Spain). Blood samples were collected in sterile sodium–heparin Vacutainer™ tubes. Lymphocyte chromosome metaphase spreads were obtained according to the de Grouchy et al. [27] technique with minor modifications [13]. Approximately 30 well-spread metaphases per animal (those with intact chromosomes and no overlapping) were examined under a Reichert Polyvar™ (Depew, NY, USA) Microscope (1250-times magnification). Subsequently, the samples that revealed chromosomal abnormalities were retested by using G-Banding [28]. Accordingly, the animals of the study were distributed into three groups: rob (1;29)-free animals (F), rob (1;29) heterozygous carriers (H_e_C), and rob (1;29) homozygous carriers (HmC).

### 2.3. Animals Tested for Genetic Diversity Studies via SNP Genotyping

To assess the genetic diversity of the populations, a total of 373 of the previously karyotyped animals (186 from BN and 187 from BC) were genotyped using an SNP microarray (Table 2). For this purpose, peripheral blood samples preserved in the CORADES Group Sample Bank at the Department of Animal Production of the University of Cordoba (Spain) were used. The sampling was carried out while attempting not to include related animals and ensuring that the groups were as homogeneous as possible (in terms of breed and sex). However, it was impossible to obtain a fully balanced sample, as there was not a sufficient number of rob (1;29) homozygous carriers.

### 2.4. SNP Genotyping and Quality Control

The processing of the samples for DNA extraction, as well as SNP genotyping, was carried out by the Xenética Fontao S.A. Laboratory (Lugo, Spain), using Illumina Bovine SNP50 v3 BeadChips and according to the manufacturer’s protocol. From this platform, we obtained information related to 58,276 SNPs.

Prior to performing calculations of genetic diversity, it was necessary to perform SNP quality selection to make the results as consistent as possible. The initial genotypes were subjected to quality filtering for which the open-access software PLINK version 1.9 [29] was used. It was carried out in two stages: in the first stage, samples from individuals with a call rate below 90% were removed from the analyses; secondly, those SNPs with a call rate below 90% or not in Hardy–Weinberg equilibrium (*p* > 10^−6^) were discarded, and only autosomal markers were considered. A threshold of 5% was applied for the minor allele frequency (MAF) filter for all the analyses with the exception of the identification of ROH regions. 

After performing quality control, a total of 369 animals were analyzed (Table 1): 184 belonging to BC, including 101 translocation-free (BCF) and 83 translocation carriers (BCC); and 185 belonging to BN, including 99 translocation-free (BNF) and 86 translocation carriers (BNC). There was a total of 55,869 autosomal SNPs for all populations.

### 2.5. Genetic Diversity Estimation and Population Structure

The genetic diversity was calculated separately for the whole samples of each Berrenda cattle breed (BN and BC) and for 4 groups established according to the presence or absence of the rob (1;29) translocation: BN translocation-free (BNF), BN translocation carriers (BNC), BC translocation-free (BCF), and BC translocation carriers (BCC). Genetic diversity values were calculated through the degree of polymorphism of the SNP markers or the average minor allele frequency (MAF) and the average proportion of SNP markers with an MAF ≥ 0.05. MAF values were calculated by using the freq function of the PLINK 1.9 software.

The observed (H_o_) and expected (H_e_) heterozygosity, Wright’s inbreeding coefficient (F_IS_), and mean inbreeding increase (ΔF) were also calculated. Inbreeding coefficients derived from runs of homozygosity (ROH) analysis, using the detectRUNS package [29], were also determined for each individual and autosome in each of the breeds and populations. The mean value (F_ROH_) was calculated considering different ROH lengths, according to the following categories: <4 Mb, from 4 to 10 Mb, from 10 to 20 Mb, and >20 Mb [30,31]. The criteria applied to detect homozygosity regions were a window size of 50 SNPs with a threshold of 0.05, allowing 1 heterozygous and 1 missing call per window at most, a maximum gap between homozygous SNPs of 1 Mb, at least 1 SNP each of 100 Kb, a minimum length to be considered as an ROH of 1 Mb, and 10 SNPs inside the ROH. These categories and criteria were selected based on different authors [31,32,33,34,35].

To characterize the population structure, the population differentiation coefficient (F_ST_) per population was calculated. In addition, a principal component analysis (PCA) was also performed. All calculations were performed using the PLINK software version 1.9 [29]. 

The difference across the genome among the four groups established according to the breeds and the presence or absence of the chromosomal disorder was assessed using the F_ST_ pairwise distance [36], which measures the variation in the locus-specific allele frequency between the different established groups. F_ST_ pairwise distances were obtained using PLINK version 1.9 [29] with the default settings.

To identify regions of the genome that could explain the differences between groups, the rolling average of the F_ST_ values was calculated for a window of 10 SNPs to account for the locus-by-locus random variation in each autosome [37].

For those genomic regions in which significant differences among breeds and/or rob (1;29) groups or homozygosity regions with high prevalence in the populations were observed, we determined the presence of genes of interest. This was conducted using the biomaRt R package [38]. Thus, the mentioned regions were mapped to the bovine reference genome, namely the *Bos taurus* ARS-UCD1.3 assembly, available in bioMart (https://www.ensembl.org/info/data/biomart/index.html, accessed on 2 November 2023).

## 3. Results and Discussion

### 3.1. Genetic Diversity Analyses

The implementation of selection strategies against rob (1;29) based on the removal of carrier sires in the populations of the Berrenda cattle breeds could lead to a reduction in genetic diversity and an increase in the levels of inbreeding. The results showed quite similar values for the populational parameters, namely the average minor allele frequency (MAF), the proportion of SNP markers with MAF ≥ 5% (%MAF05), the number of polymorphic loci (N_P_), and the percentage of polymorphic loci (N_P_%). No significant differences (*p* > 0.05) between the two Berrenda cattle breeds and groups were found for the observed heterozygosity (H_o_), but the differences were significant (*p* > 0.05) for the expected heterozygosity (H_e_) and Wright’s Individual Variability Index (F_IT_). Moreover, in previous studies with DNA microsatellites, the genetic diversity was higher in the BN breed compared to the BC breed [25] because of the larger effective population size in BN.

In the present study, the results did not clearly indicate that the presence of rob (1;29) had an effect on the conservation of the genetic variability, although the individuals of the two translocation-free populations (the BCF and BNF groups), which could be used as breeding animals, showed slightly lower genetic variability than those belonging to the translocation carrier populations (the BCC and BNC groups), according to the genetic diversity parameters analyzed (Table 3). Only in the case of the Berrenda en Negro cattle breed did the translocation-free population (the BNF group) retained higher observed heterozygosity (H_o_) than the translocation carrier population (the BNC group). This suggests that the removal of the carrier populations could lead to a certain loss of genetic variability, and therefore, the eradication programs should be progressive.

The MAF values (the frequency of the less common allele in a population) ranged from 0.200 in the BNF group to 0.206 in the BCC group, being very similar in all the established groups. The MAF values were lower than those obtained in other red coat cattle breeds from northern Europe whose values ranged from 0.222 to 0.281 [39]. These differences in MAF values could be explained by the small population size of the Berrenda cattle breeds, which is a typical feature of any threatened breed. However, these values were consistent with others reported in Italian minor cattle breeds such as Mucca Pisana and Sardinian (MAF = 0.200–0.267) [40]. In contrast, the obtained values were lower than those reported by Matukumalli et al. [41] in 14 different cattle breeds with a larger population (MAF = 0.240–0.310). 

The proportion of SNPs with MAFs ≥ 0.05 was high, ranging from 70.3% in the BCF group to 71.7% in the BCC group. This proportion was lower than that obtained in other Spanish native cattle breeds with similar livestock production systems (beef cattle under extensive farming and practicing natural mating), such as Asturiana de los Valles (89.28%) and Bruna dels Pirineus (88.17%) [42]. However, it is important to highlight that these values were estimated from 735,239 SNPs, a much larger number of markers compared to our study (58,276 SNPs), and the Berrenda cattle breeds were likely not perfectly represented in the chips, i.e., not all markers in the chip were actually polymorphic in these two breeds as they were not taken into account when designing the chip. In the case of the Rubia Gallega cattle breed, 72.1% of the markers showed MAF values ≥ 0.01 when using the Axiom Bov MD_v3 chip (Affymetrix™, Santa Clara, CA, USA), which was based on 57,053 SNPs [43]. 

In another geographical area, studies on Czech cattle showed a proportion of SNPs with MAFs ≥ 0.05, which is higher than in the Berrenda cattle breeds (92.6–94.32%) [37]. In other studies, different European cattle breeds showed similar degrees of polymorphism as the Berrenda cattle breeds (63–83%), while certain African breeds presented a lower interval than the one obtained in our study (47–71%) [44]. 

Regarding the heterozygosity or genetic diversity within the groups, the observed heterozygosity (H_o_) ranged from 0.300 in the whole population of BN and BC to 0.313 in the BNF group. Meanwhile, the expected heterozygosity (H_e_) presented a value of 0.323 in the whole BN population and 0.336 in the BCC group. The observed and expected heterozygosity in the total population of the Berrenda cattle breeds were lower than the values obtained in the Rubia Gallega breed (0.336) [43] and similar to the values obtained in a different study carried out on seven Spanish cattle breeds, where H_o_ varied from 0.299 to 0.319 [42]. Considering red cattle populations from northern Europe, only the Traditional Danish Red breed showed H_o_ mean values very similar to those obtained in the Berrenda Spanish cattle breeds, while the remaining ten breeds of the mentioned study presented higher values. Regarding the mean values, the majority of these ten breeds showed higher values than the BN and BC breeds, but the Traditional Danish Red breed and the Groningen White Headed breed showed lower values. The Dutch Belted breed (H_e_ = 0.323) presented values very similar to BN, and the Dutch Red Friesian breed (H_e_ = 0.337) obtained values very similar to BC [39]. In the case of Czech cattle, they showed higher H_e_ and H_o_ values than the Berrenda cattle breeds (H_e_ = 0.346–0.356 and H_o_ = 0.362–0.377) [37].

The H_o_ and H_e_ calculations based on SNP analysis yielded much lower values than those based on microsatellite analysis in the Berrenda cattle breeds [27]. This could be explained by the fact that calculations of these two parameters tend to be overestimated when they are based on microsatellites in comparison with calculations based on SNPs [45,46]. 

H_e_ was lower than H_o_ in all the groups established in both Berrenda breeds, which indicates a certain degree of inbreeding or the presence of a form of subdivision at the population level, possibly due to genetic drift or to the Whalund effect, so that gene flow was hindered by geographical barriers or by non-random mating [3,21]. In a recent research work based on analyses of genealogical registers in the Berrenda cattle breeds [3], the lack of exchange of breeding animals among herds was revealed: 25% of BN breeders and 20% of BC breeders do not provide bulls to other farms to be used as breeding males. Although they purchase sires from other farms, approximately 30% of sires are born and raised in the farms themselves. Currently, Berrenda breeders’ implementation of reproductive management tools such as artificial insemination or planned exchanges of breeding stock between herds is still scarce.

A similar selection program against Robertsonian translocation rob (1;29) has been carried out in the Spanish Retinta cattle breed, based on the removal from the breeding stock of bulls carrying this chromosomal disorder (both homozygous and heterozygous carriers), without causing any significant reduction in genetic variability at the breed level [12]. Although our results show that the Berrenda cattle breeds retain a large amount of their ancestral genetic variability, the strong selection pressure during the first few years of the implementation of the rob (1;29) eradication plan (in the case of BN, the incidence of carriers was reduced from 47 to 11% in only 10 years [17]), together with the scarce breeding stock exchange between herds, could explain the differences in the levels of polymorphism found in these two threatened breeds in comparison with other European minor breeds. 

In general, independently of whether the animals are rob (1;29) carriers or not, this study does not reveal notable differences in the genetic variability within breeds. Nonetheless, in the case of the BC breed, the estimations indicate that rob (1;29) carriers possess slightly higher genetic variability than rob (1;29)-free individuals. 

### 3.2. Analyses of Inbreeding and Runs of Homozygosity (ROH)

Inbreeding monitoring is especially appropriate in low-census breeds that are inherently susceptible to genetic drift, as a continuous increase in inbreeding within a population is unavoidable if the number of breeding animals is finite. The decrease in the number of heterozygotes is accompanied by inbred depression for economically important traits, but it also reduces the possibilities of adaptation to unforeseen future changes (e.g., changes in means of production, climatic changes, animal health policies, etc.). For this reason, genetic diversity studies are of great relevance particularly with regard to endangered populations. 

The F_IS_ parameter was positive, which is indicative of the deficit of heterozygotes. F_IS_ was higher in the BC breed (Table 4), which is consistent with the increase in inbreeding per generation, which was higher in BC than BN (0.101 vs. 0.077). Regarding the different population groups considered, BCC and BNF were the groups that showed the lowest F_IS_ values and the lowest increases in inbreeding per generation, which is in accordance with the highest heterozygosity values previously mentioned. Inbreeding studies based on microsatellite analysis on the Berrenda breeds showed an excess of heterozygotes [25]. In different Spanish cattle breeds, positive F_IS_ values were reported [42].

Genome-wide runs of homozygosity (ROH) provides useful information to understand the genetic relationships between individuals, which is useful to minimize the rate of inbreeding and to identify harmful genomic variations [47]. 

**Figure 4:**
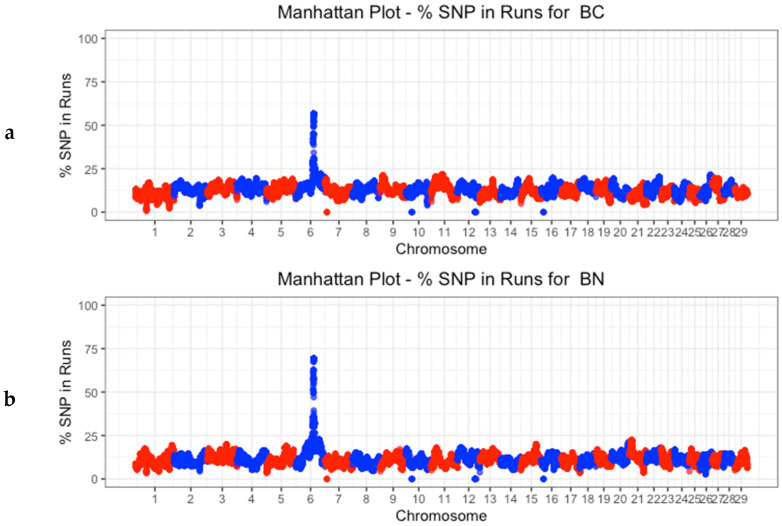
Manhattan plot of the percentage of times that each SNP belongs to an ROH (%SNP in ROH) by chromosome for (**a**) Berrenda en Colorado (BC) and (**b**) Berrenda en Negro (BN).

Long runs of homozygosity (ROHs) arise from recent inbreeding, while shorter ones are indicative of more distant ancestral effects, such as founder effects [48]. In fact, the presence of ROH segments larger than 10 cM might result from a common ancestor five generations in the past [49]. Concerning the populations under study, the inbreeding appears to be recent, according to the predominance of long runs of homozygosity in most of the individuals (Table 4). This fact suggests that many of them are descended from common ancestors and that they are the result of a recent inbreeding process. By analyzing the runs of homozygosity on the autosomal genome in the Berrenda breeds (Figure 4), a clear region of homozygosity was identified on chromosome 6, consistent with the location of the *KIT* gene, which is involved in determining the spotted coat pattern typical of these two breeds [50].

The program dedicated to reducing genetic diversity losses and avoiding an increase in inbreeding in the Berrenda breeds is carried out at the herd level by the farmers themselves, who avoid inbred mating, by using the technical information provided by the breeders’ associations [16]. This strategy appears to be successful for the maintenance of genetic diversity; however, the data collected suggest that these actions have not been sufficient to delay inbreeding. Pedigree analyses estimated an increase in inbreeding per generation in BC and BN of 3.5 and 4.5%, respectively [3], which are lower than the inbreeding rates obtained in this study. This may be because SNP genotyping has only been carried out on a part of the population but also because the source of information (SNPs) used to calculate inbreeding in this work was different. It is reported that genealogical and molecular inbreeding estimates may not be of the same magnitude [51].

### 3.3. Analysis of the Structure of the Population

The analysis of the Fixation Index (F_ST_), used to measure the differentiation of the population due to the genetic structure, showed that the genetic differences were generally small, ranging from 0.012 to 0.031, with low levels of genetic diversity between groups (Table 5). The smallest differences were observed between rob (1;29) carriers and non-carriers in both breeds (BN = 0.012; BC = 0.013). Conversely, the highest F_ST_ value (0.031) was observed between the two translocation-free groups (BNF and BCF), showing that 3.1% of the genetic variability was attributable to the breed and that there were greater genetic differences between breeds than between groups, which were derived from the rob (1;29) genotype. These results suggest that neither the selective actions carried out against the Robertsonian translocation nor the negative effects caused by this genetic disorder on the animals’ reproductive performance gave rise to fragmentation within the BN and BC breeds due to rob (1;29). The reproductive management implemented to avoid inbreeding also appeared to be successful. Similar pairwise F_ST_ values have been reported in Italian local cattle breeds—specifically, between the Sarda and Sardo-Bruna breeds and between the Sarda and Piedmontese breeds [40].

The principal component analysis (PCA) showed that 17 components accounted for 90% of all variation. Among them, the first three principal components explained 30% of the variation, while the first and second main components (PC1 and PC2) explained 11.02% and 10.5% of the variation, respectively.

**Figure 5:**
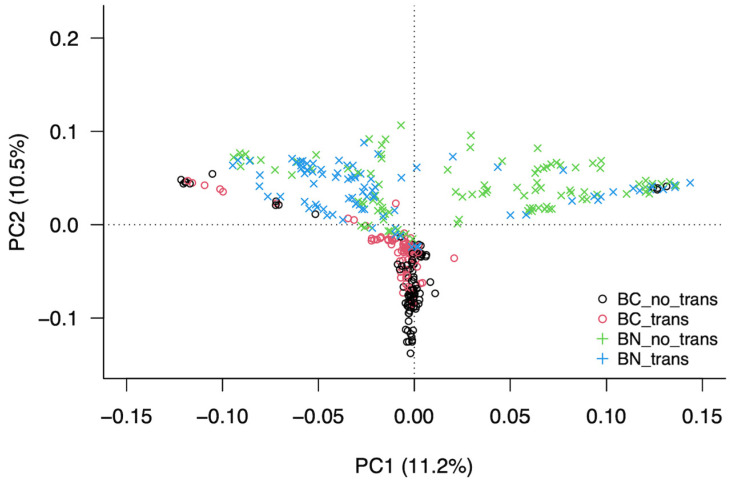

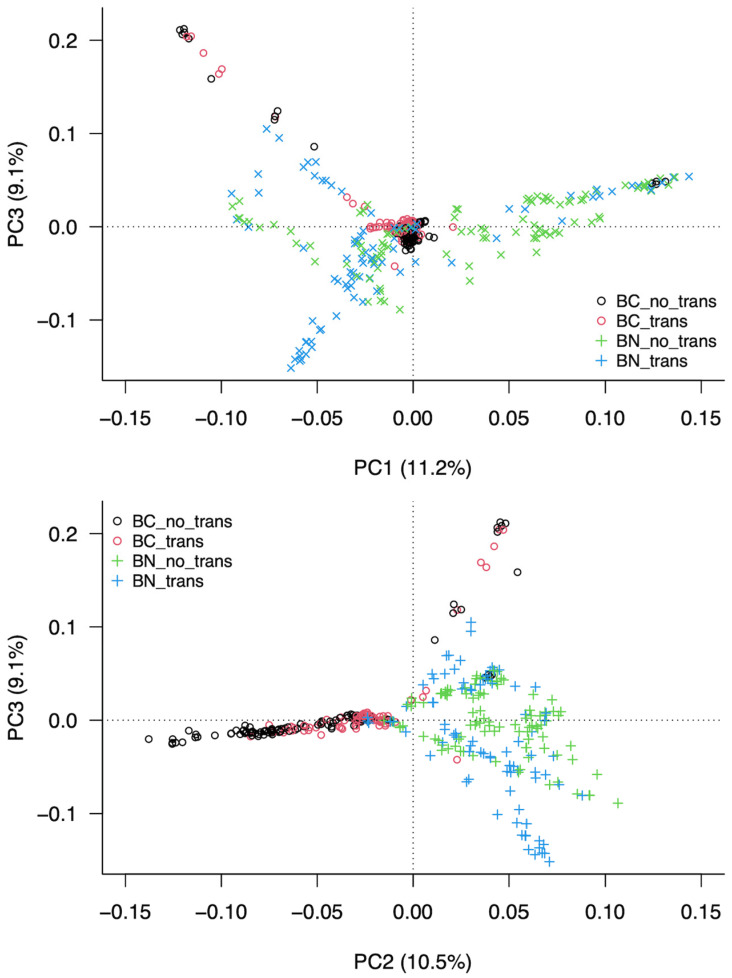
PCA plot showing the population structure of 369 genotyped animals of the Berrenda cattle breeds. Different colors and symbols separate the animals by breed and group: Berrenda en Colorado translocation-free (BC_notrans; N = 101; black circles); Berrenda en Colorado translocation carriers (BC_trans; N = 83; red circles); Berrenda en Negro translocation-free (BN_notrans; N = 99; blue); and Berrenda en Negro translocation carriers (BN_trans; N = 86; green).

Figure 5 shows the distribution of the animals considering only the first three main components (PC1, PC2, and PC3). PC2 was the component that best discriminated between the two breeds regardless of whether the animals were rob (1;29)-free or rob (1;29) carriers. Most of the BC animals were grouped together and overlapping for the PC1 and PC3 components, and they were located separately from BN animals along the PC2 component.

In the case of the BN breed, some variation was detected between the two karyotyped groups. PC1 was the component that best explained the observed variation, separating some of the rob (1;29) carriers from the rob (1;29)-free individuals.

In the present study, the PCA results were consistent with the pairwise F_ST_ values between the BC and BN populations, which suggests that part of the genetic diversity was shared by the two breeds, since a large proportion of the SNPs were found in both populations, which was possibly due to the genetic contributions of their common ancestors. Nevertheless, a significant number of SNPs (1477 in BC and 655 in BN) were fixed in one of the breeds but not in the other, which reflects their genetic uniqueness. Both breeds varied widely not only due to the different origins of each of their founders but also because of the reproductive isolation that they had experienced for more than five generations as well as the coat-color-selective strategies implemented to establish and retain their phenotypic distinctiveness [3]. Because of their phenotypic similarity, these two cattle breeds were for a long time considered as one, which resulted in frequent mating between animals of the two breeds [4]; however, the outcomes of this work indicate that nowadays, the genetic differences between the two Berrenda breeds are due to the reproductive isolation that occurred between the two populations once their herd books were established separately.

Our PCA results suggest the possibility of identifying, through SNP, regions of the genome associated with the alteration rob (1;29). Recently, Iannuzzi et al. [52] identified a new genomic biomarker that allows the identification of rob (1;29) carriers in a faster and cheaper manner than the classic cytogenetic techniques.

### 3.4. Identification of Discriminatory Regions between Populations along the Genome

**Figure 6:**
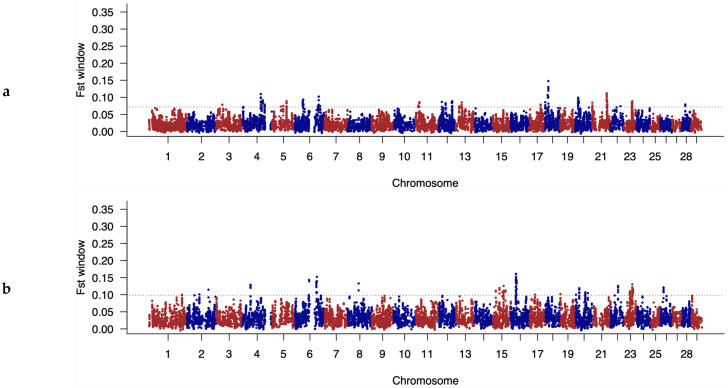
Manhattan plot representing F_ST_ values for each SNP along the autosomal chromosomes for (**a**) the rob (1;29) carrier population and (**b**) the rob (1;29)-free population in the two Spanish Berrenda cattle breeds (BN vs. BC). The value displayed corresponds to the rolling average for a window of 10 SNPs. The chromosomes are alternately colored in red and blue. The dotted line represents 0.1% of the top FST observations.

A genome-wide association analysis was carried out on the SNP dataset from all the animals in order to identify breed markers in the population carrying rob (1;29) and the population free of this Robertsonian translocation. Figure 6a,b show the genetic diversity at the individual level (F_ST_) of all the individuals belonging to both Berrenda breeds, considering a window of 10 markers in every pair of chromosomes. 

The F_ST_ values identify those regions of the genome along the autosomal chromosomes that are highly differentiated between groups. The dotted line indicates the limit over which these markers are located above the 1% highest F_ST_ value, which is the criterion established in this work to identify genetic markers potentially associated with genetic diversity.

When comparing the two groups, the group of rob (1;29) carriers and the group free of this genetic disorder, the regions that showed the most significant differences in terms of SNPs between the two Berrenda breeds were those located in autosomal chromosomes BTA-4, BTA-6, BTA-18, and BTA-22. These regions aligned with the locations of some of the genes involved in the phenotypic characteristics of the Berrenda breeds: the melanocortin receptor 1 (*MC1R*) gene (locus Extension), which controls the coat color in cattle through the production of black and red pigments, is located in BTA-18 [50]; the *KIT* proto-oncogene, a receptor tyrosine kinase (*KIT*) gene, which is reported to be responsible for the dominant white coat color and the spotted coat pattern in cattle, is located in BTA-6 [53]; and the melanocyte inducing transcription factor (*MITF*) gene, which clearly explains the differences between spotted and non-spotted coat phenotypes (Piebald pattern) in Holstein and Simmental cattle breeds, is located in BTA-22 [54]. Additionally, the melanocortin 1 receptor (*MC1R*) and *KIT* genes have been identified as positive ancestral selection signals linked to the domestication process [55,56]. These findings are consistent with the coat color differences that distinguish both Berrenda breeds due to the breeders’ preferences for specific spotted coat patterns, such as the “red hood” (“capirote”) in the BC breed and the “black-sided” coat (“aparejado”) in the BN breed [4]. Our results suggest the possibility of providing a pool of markers to the National Association of Berrenda Cattle Breeders (ANABE) to be used as a control tool to guarantee the authenticity of their products.

This study provides valuable information to the National Association of Berrenda Cattle Breeders (ANABE) for the decision-making process regarding the implementation of selective strategies against rob (1;29).

According to the National Association of Retinta Cattle Breeders, the incidence of rob (1;29) reached 32% in 1989; since then, they have implemented a program to address this genetic disorder based on the analysis and detection at the farm level of all homozygous and heterozygous carriers and the subsequent elimination of all breeding bulls carrying the translocation from the breeding stock while retaining all the female carriers as breeding animals. Thanks to the implementation of these measures, the incidence of rob (1;29) in the Retinta cattle breed was significantly reduced by up to 6–7% in 2013 [57], reaching an incidence rate of only 1% in 2021 [12]. 

A reduction in the incidence of rob (1;29) would contribute positively to the conservation of threatened cattle breeds not only by improving their reproductive performance but also by increasing their effective population size in the medium and long term [12,57].

Considering that the Berrenda breeds are threatened breeds, and taking into consideration the higher genetic variability found in the present work in the populations affected by rob (1;29) in comparison with the rob (1;29)-free populations, the strategy implemented in the two Berrenda breeds should be gentler than the one applied in the Retinta breed. Consequently, the following measures are proposed to be implemented in the Berrenda cattle breeds: (i) the elimination of all homozygous male carriers from the breeding stock; (ii) the mating of heterozygous carriers with rob (1;29)-free animals; (iii) the implementation of appropriate genealogic control in all future breeding stock; and (iv) the use of suitable and easy-to-implement methods for the diagnosis of rob (1;29) in the candidates for the breeding stock and their ascendants.

## 4. Conclusions

The two Spanish Berrenda cattle breeds currently retain remarkable genetic diversity, but selective strategies for the elimination of the Robertsonian translocation rob (1;29) should be carried out progressively and in a controlled manner, especially in the Berrenda en Colorado breed, because of the smaller number of registered animals and also the fact that the population carrying rob (1;29) in this breed shows higher genetic variability.

The presence of rob (1;29) has not resulted in associated sub-structuring within each of the Berrenda breeds. The differences between these two breeds are more evident than those found between the subpopulations defined by the presence or lack of rob (1;29) carriers.

The identification of the autosomal regions that establish the differences between the two breeds suggests that they are associated with those genes that determine the differentiating characteristics between the two breeds based on the phenotypic coat traits.

The improvement in the reproductive performance of the Berrenda breeds requires the implementation of breeding strategies based on the contributions to the populations of the breeding stock carrying the Robertsonian translocation rob (1;29).

## Figures and Tables

**Table 1:** Number of animals sampled by sex and herd from each location.

	Berrenda en Colorado			Berrenda en Negro		
Region	Males	Females	Farms	Males	Females	Farms
Castilla y León	6	29	8	13	7	4
Madrid	14	3	7	11	3	8
Extremadura	33	2	7	25	1	3
Castilla La Mancha	2	15	4	7	31	10
Andalucía	39	42	15	36	51	16
Comunidad Valenciana	0	2	1	1	2	2

**Table 2:** Number of genotyped animals using SNP microarray, according to rob (1;29) karyotyping results, in the Berrenda en Colorado (BC) and Berrenda en Negro (BN) cattle breeds. Free = rob (1;29)-free animals; HeC = rob (1;29) heterozygous carriers; HmC = rob (1;29) homozygous carriers. Number of animals considered after quality control is indicated in brackets.

Animals	BC				BN			
	Total	Free	HeC	HmC	Total	Free	HeC	HmC
Females	93	51	38	4	94	30	63	1
Males	94	53	37	4	92	70	22	
Total	187 (184)	104 (101)	75 (75)	8 (8)	186 (185)	100 (99)	85 (85)	1 (1)
Farms	42	14	28	7	43	11	32	1

**Table 3:** Indicators of genetic diversity obtained by SNP analysis in the Berrenda en Negro (BN) and Berrenda en Colorado (BC) cattle breeds and in the four subpopulations determined by the presence or absence of rob (1;29) carriers.

Group	*n*	MAF	%MAF_05_	N_p_	N_p_%	F_IT_ (±SE)	H_o_ (±SE)	H_e_ (±SE)
BC	184	0.205	71.5%	44,129	81.5	0.09 (0.01) ^A^	0.306 (0.01)	0.334 (0.00) ^A^
BN	185	0.203	70.9%	44,951	83.0	0.09 (0.01) ^A^	0.308 (0.01)	0.328 (0.00) ^B^
BCF	101	0.201	70.3%	43,096	79.6	0.12 (0.01) ^B^	0.302 (0.01)	0.333 (0.00) ^B^
BCC	83	0.206	71.7%	43,720	80.7	0.08 (0.01) ^A^	0.311 (0. 01)	0.336 (0.00) ^A^
BNF	99	0.200	70.4%	43,678	80.7	0.09 (0.01) ^A^	0.313 (0.01)	0.328 (0.00) ^D^
BNC	86	0.202	70.8%	43,990	81.2	0.09 (0.01) ^A^	0.304 (0.01)	0.329 (0.00) ^C^

*n*: sample size; MAF: average minor allele frequency; %MAF_05_: proportion of SNP markers with MAF ≥ 5%; H_o_: observed heterozygosity; H_e_: expected heterozygosity; N_p_: number of polymorphic loci; N_p_%: percentage of polymorphic loci; F_IT_: methods-of-moment inbreeding; BC: Berrenda en Colorado breed; BCF: BC rob (1;29)-free group; BCC: BC rob (1;29) carrier group; BN: Berrenda en Negro breed; BNF: BN rob (1;29)-free group; BNC: BN rob (1;29) carrier group. ^A–D^ Different superscripts indicate significant differences (*p* < 0.05) between breeds and among four subpopulations for the F_IT_, H_o_, and H_e_ parameters obtained from an ANOVA test.

**Table 4:** Mean values of the inbreeding coefficients in Berrenda en Negro (BN) and Berrenda en Colorado (BC) cattle breeds and in the populations determined by the Robertsonian translocation rob (1;29), estimated via Wright’s inbreeding coefficient (F_IS_), ΔF (increase in inbreeding), and the inbreeding coefficients derived from runs of homozygosity (FROH) for different length categories.

Group	*n*	F_IS_ (±SD)	ΔF	F_ROH_ (±SD)	F_ROH_ < 4 Mb (±SD)	F_ROH_ < 10 Mb (±SD)	F_ROH_ 10–20 Mb (±SD)	F_ROH_ > 20 Mb (±SD)
BC	184	0.09 (0.13)	0.101	0.13 (0.12)	0.01 (0.008)	0.03 (0.02)	0.04 (0.03)	0.09 (0.079)
BCF	101	0.09 (0.13)	0.100	0.14 (0.12)	0.01 (0.009)	0.03 (0.02)	0.05 (0.03)	0.010 (0.08)
BCC	83	0.08 (0.13)	0.082	0.11 (0.12)	0.01 (0.008)	0.02 (0.02)	0.04 (0.03)	0.09 (0.08)
BN	185	0.07 (0.12)	0.077	0.12 (0.13)	0.01 (0.009)	0.03 (0.02)	0.04 (0.03)	0.08 (0.06)
BNF	99	0.05 (0.13)	0.053	0.10 (0.11)	0.01 (0.01)	0.03 (0.02)	0.04 (0.03)	0.08 (0.07)
BNC	86	0.08 (0.12)	0.084	0.13 (0.11)	0.01 (0.01)	0.03 (0.02)	0.05 (0.03)	0.08 (0.06)

*n*: number of animals; SD: standard deviation; BC: Berrenda en Colorado breed; BCF: BC rob (1;29)-free group; BCC: BC rob (1;29) carrier group; BN: Berrenda en Negro breed; BNF: BN rob (1;29)-free group; BNC: BN rob (1;29) carrier group.

**Table 5:** Genetic differentiation values (F_ST_) between rob (1;29) carriers and rob (1;29)-free animals in the Spanish Berrenda cattle breeds.

Group	BCC	BNC	BNF
BCF	0.013	0.028	0.031
BCC		0.021	0.026
BNC			0.012

BCF: BC rob (1;29)-free group; BCC: BC rob (1;29) carrier group; BNF: BN rob (1;29)-free group; BNC: BN rob (1;29) carrier group.

## Data Availability

This is not applicable, as the data are not included in any data repository with public access. However, if the readers require access, the authors will happily provide this; please email pa1rosee@uco.es.
